# Histomorphogenesis of cranial nerves in *Huso huso* larvae

**Published:** 2016-06-15

**Authors:** Sherma Tavighi, Zohreh Saadatfar, Bahador Shojaei, Morteza Behnam Rassouli

**Affiliations:** 1 *Department of Basic Sciences, Faculty of Veterinary Medicine, Ferdowsi university of Mashhad, Mashhad, Iran; *; 2 *Department of Basic Sciences, Faculty of Veterinary Medicine, Shahid Bahonar University of Kerman, Kerman, Iran; *; 3 *Department of Physiological Sciences, Faculty of Biology, Ferdowsi university of Mashhad, Mashhad, Iran.*

**Keywords:** Brain nerves, Development, Histology, *Huso huso*, Larvae

## Abstract

In this study the cranial nerves development of *H. huso* are explained from 1 to 54-days-old (1, 3, 6, 15, 21 and 54 days). Despite all the researches on fish brain, there are no study on nerves evolution on* H. huso *during their larvae life. For this research 40 samples of larvae *H. huso* were obtained (from each age, about six samples were selected). The specimens were maintained in fiberglass tank, then histological samples were taken from tissues and stained with hematoxylin and eosin for general histological studies using light microscope. According to the results, on 1 and 3-days-old, no nerve was observed. The terminal nerve and their dendrites were observed around the nasal cavity and the axons projected to different areas in forebrain especially around olfactory bulb diffusely, on 6-day-old fish. Also, olfactory, optic, oculomotor, trochlear, trigeminal, lateral line and vagus nerves were detected on 6-day-old fish, however two parts of lateral line nerve were separated on 54-day-old. Three nerves, profundus, facial and octaval were observed on 54-day-old, however, up to this age, epiphysial nerve was not observed.

## Introduction

The sturgeons are most valued fishes in Caspian Sea and *H. huso* is one of the most long life sturgeon species.^1^ They are pelagic fishes which the adults usually live in middle depths sea and these fishes do not live in sea bottom. However, they require rather low light environments and use of special senses for life activities like the ability of their perception of earth magnetic circuit to navigate their migration path without using other environmental factors.^1^ Other sturgeon species are normally benthic and swimming bottom sea.^2^

Variation of ray-finned fish’s brain structure happens independently during development^3^ and it is reflective of key points in relation to evolution of nerves.

Despite all the researches on fish brain, there are few studies on nerve development. The primary projection of the trigeminal nerve was studied in 1984 on *Acipenser oxyrhynchus* and *Acipenser scaphirhynchus*. However, there is no study on nerves evolution on *H. huso* during their larvae life.

Nerve fibers in actinopetrygians are responsible for different behaviors and senses such as vision, gustatory, smell, balance and eye movements.^4^ The nerves may be sensory and carry impulses to the different areas of brain, or may be motor and carry information from brain to other structures of body, and finally they may be mixed (motor and sensory).^4^

Sensory system is classified into chemical senses (i.e. taste and smell), mechanical senses (i.e. hearing and touch) and electromagnetic senses (i.e. visual and electroreceptive).^5^

Motor system is classified into: dorsal, ventral and parasympathetic parts, similar to sensory system, that innervate various areas. In ray-finned fishes like catfishes motor systems especially V nerve,^5^ distinguish edible stuff in the near water and produce the necessary suction to ingestion of food.^5^ The sensory and motor partitions of nerves are fragmentally formed and the positions which they enter or exit the brain reflect their developmental period.^5^ In most of fishes, all the brain nerves are identifiable and contain 22 nerves. The first four nerves are in the forebrain and entirely are sensory including: terminal (Te), olfactory (I), optic (II) and epiphysis (Ep) nerves.^6^ Midbrain includes two motor nerves of: oculomotor (III) and trochlear (IV).^6^ The remaining cranial nerves originate from hindbrain. All fishes lacked XI and XII nerves. Moreover, Huge fishes do not have the optic and epiphysis nerves.^7^ The nerves in all vertebrates have the same names and functions.^7^ Oculomotor nerve is the somatic motor nerve of eye muscles and innervates the extrinsic extra orbital eye muscles.^7^

The trigeminal nerve (V) arises from the anterior end of the medulla. It is a mixed motor and sensory nerve which has four branches that innervate the face, eyes, mouth and jaws.^6^ The superficial ophthalmic nerve is of the four branches. It has a general sensory function for the skin of the rostrum. Maxillary branch innervates superior jaw and mandibular branch innervates the structures of mandible and tongue. The most of fishes profundus nerve (Pro) innervates to the mucosal layer of lateral line.^6^

The vagus nerve (X) is the longest of the cranial nerves. It is a mixed motor and sensory nerve that arises at the posterior end of the medulla. It innervates the gills, throat, esophagus, stomach, intestines and body wall.^6^

Fishes and other aquatic animals bear the mechano-sensory lateral line. This structure is located on the surface of the head and the body. The sturgeons similar to other fishes have six detached nerves including: anterio-dorsal, anterio-ventral, otic, middle, supra-temporal and posterior lateral line nerves. Amphibian ambystoma has five lateral line nerve and lacks otic nerve.^4^

The highly economic importance of *H. huso* is for the caviar,^8^ and also It is valuable meat. This study illustrated cranial nerves morphogenesis in *H. huso* larvae from 1 to 54-days of age. 

## Materials and Methods

For this research we obtained 40 samples of *H. huso* larvae with total average weight of 0.81 g and length of 2.03 cm. The ages of 1, 3, 6, 15, 21 and 54 days post hatching (dph) were obtained from Shahid Marjani's Agh ghala propagation station, Gorgan, Iran. The specimens were maintained in fiberglass tank. At first morphometric parameters like total length and weight were measured caliper and digital balance device (Model 47257 SKU; Pittsburgh^®^, Camarillo, USA), then Six larvae of each age were fixed in 10% buffered formalin (Merck, Darmstadt, Germany) in 48 hr, dehydrated with ethanol (Merck) series 50% to 100%, cleared in xylene (Merck) and embedded in paraffin (Merck) by tissue processing device (Pouyan Teb Khadem, Mashhad, Iran). Because the brain was very small in size, total head was fixed and embedded in paraffin blocks. We used lockhart's molds for making paraffin blocks. Blocks were cut frontally and sagittally in serial sections and ribbon shaped with regular intervals into 6 µm thickness from the primary specimens to ends by using an auto cut rotary microtome (Leica, Wetzlar, Germany).^3^^,^^5^^,^^7^^,^^9^ Tissue sections were deparaffinized and stained with hematoxylin and eosin (H & E) and about 10 sections from each specimens were selected and observed by a camera attached to the light microscope (Model CX22; Olympus, Tokyo, Japan), with 200× and 400× magnifications for general histological studies.

## Results

According to the results of the current research, on 1 and 3-day-old fishes, the cranial nerves were not observed in *H. huso* larvae. Histomorphogenesis of cranial nerve in *H. huso* larvae began on 6-day-old fish.

**Fig. 1:**
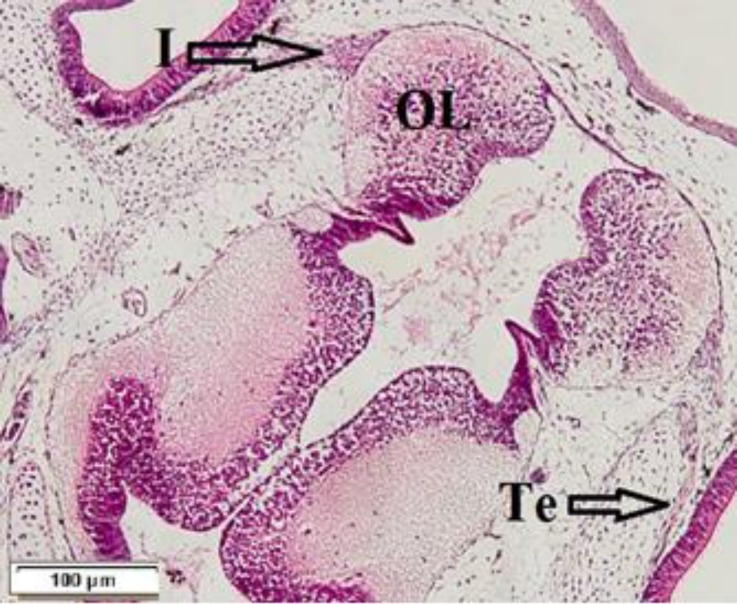
Frontal section of the brain from 6-day-old, OL: olfactory bulb, I: olfactory nerve, Te: terminal nerve (H & E

On 6-day-old fish, the Te nerves and their dendrites were observed around the nasal cavity and the axons were projected to different areas in forebrain especially around olfactory bulb diffusely (Fig. 1). The olfactory nerve (I) was branched from the olfactory placode and was pairwise. The nerve carried olfaction formations to olfactory bulb at the rostral part of telencephalon and was distinguished on 6-day-old fish and with the growth of larvae became thicker. The nerve was formed from appendices of efferent bipolar olfactory cells (Fig. 1).

The optic nerve (II) was large and originated from the neurons in retina and was pairwise. This nerve was located caudo-medial from the surface of orbit eye in *H. huso* and detected on 6-day-old fish. With growth of larvae this nerve was larger and in chiasma optic coursed each other. Epiphysial nerve (Ep) was not distinguished up to 54-days in *H. huso*. Oculomotor nerve (III) exited from the middle tectum nearby ventro-medial midbrain and was observed on 6-day-old fish.

**Fig. 2:**
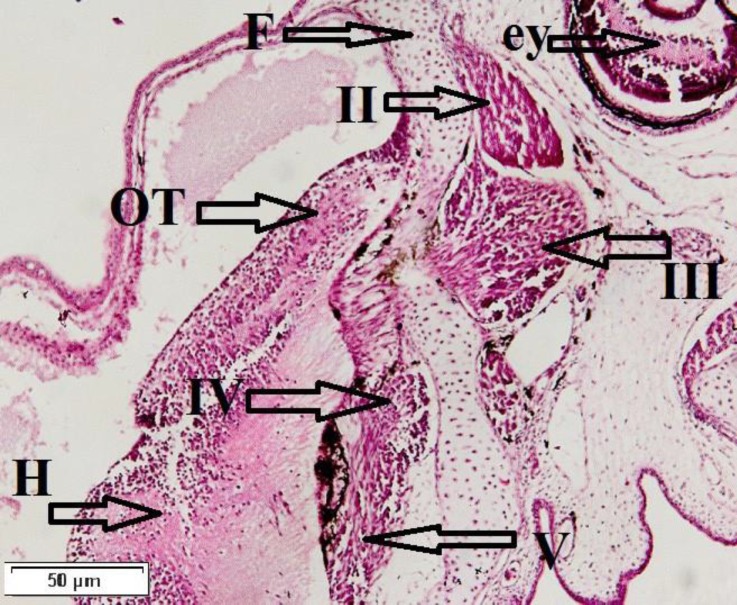
Sagittal section of the brain from 6-day-old, OT: optic tectum, F: forebrain, H: hindbrain, II: optic nerve, III: oculomotor nerve, IV: trochlear nerve, V: trigeminal nerve, ey: eye (H & E

Trochlear nerve (IV) was the only nerve exited from the caudo-dorsal area of midbrain and innervated upper oblique of the eye muscle of the opposite side and was detected on 6-days in *H. huso* larvae (Fig. 2).

**Fig. 3.**
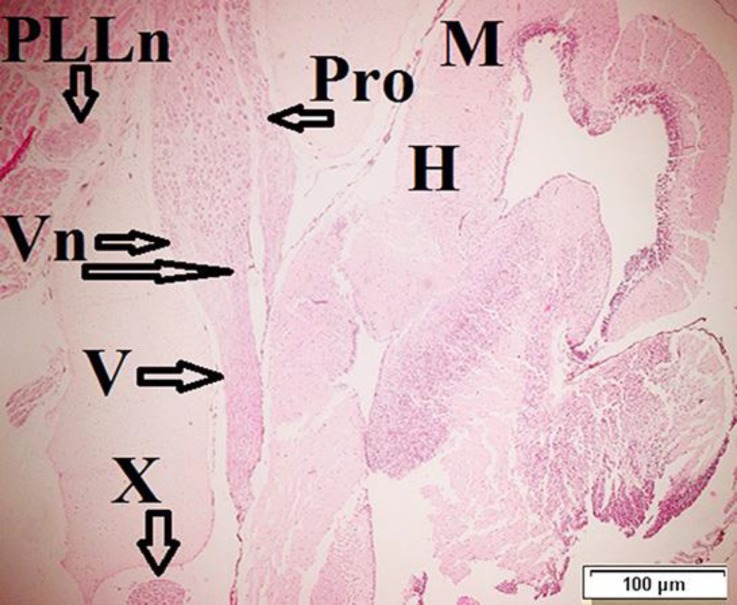
Frontal section of the brain from 54-day-old, V: trigeminal nerve, H: hindbrain, M: midbrain, Vn: trigeminal nucleus, Pro: profundus nerve, X: vagus nerve, PLLn: posterior lateral line nerve (H & E

The Pro nerve was very small. It is a sensory component. The Pro nerve exited from rostral part of the hindbrain and was located toward snout. The Pro nerve was distinguished on 54-day-old fish (Fig. 3).

Trigeminal nerve (V), originated bilaterally from caudo-ventral of hindbrain and lied on toward rostrally area of brain on 6-day-old (Fig. 2). Two nuclei were observed in this area: trigeminal sensory and descending trigeminal stripe nuclei. These nuclei were located in rostro-caudal area and formed long cell sensory columns on 54-day-old fish (Fig. 3).

**Fig. 4.**
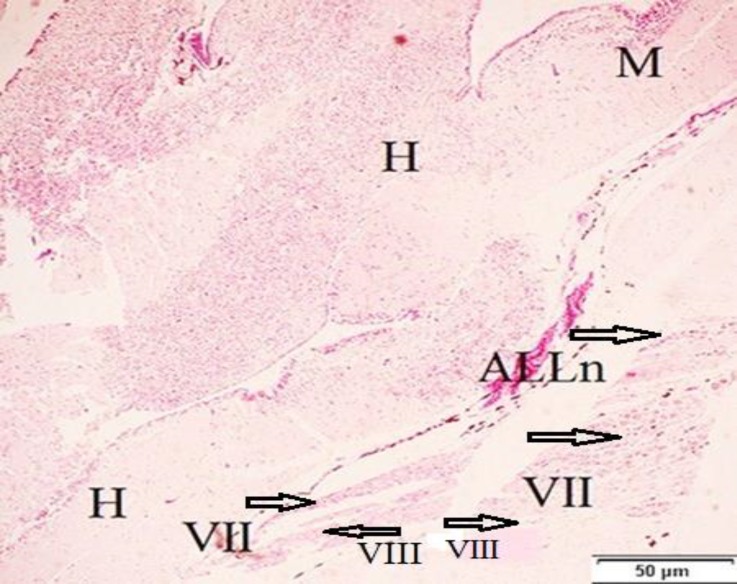
Frontal section of the brain from 54-day-old, H: hindbrain, M: midbrain, VII: facial nerve, ALLn: anterior lateral line nerve, VIII: octaval nerve (H & E

Lateral line nerve (LLn) contained anterior lateral line (ALLn) and posterior lateral line (PLLn). The LLn was observed without any separation bilaterally in rostro-dorsal, rostro-ventral and inner otic area on 6-day-old fish. The ALLn was located in the ventro-medial part of the inner ear (Fig. 4). The PLLn was located in the dorso-caudal part of the vagal lobe and both two parts were separately detected on 54-day in *H. huso* larvae (Fig. 3).

Facial nerve (VII) was located in rostro-ventral part of the medulla oblongata and observed on 54-day-old fish (Fig. 4). It formed cranial part of the facial-octaval ganglion complex. The central root of the facial nerve entered the hindbrain in a similar manner to position of their motor root. 

Octaval nerve (VIII) formed the dorso-caudal part of the facial-octaval ganglion complex and originated from hindbrain on 54-day-old fish (Fig. 4) and entered to the lateral surface of the brain stem and was related with inner ear.

Vagus nerve (X) was a complex nerve with some branches and located in the caudal part of hindbrain. It was observed on 6-day-old fish and with aging up 54-day became thicker in *H. huso* larvae (Fig. 3).

## Discussion

Development of the nerves in H. huso larvae was shown in this research. Comparison between in the previous and other reports in vertebrates shows a series of conflicts.^9^^,^^10^ The axons of olfactory nerve in some of the birds and bony fishes, are the shortest in the cranial nerve axons.^9^^,^^10^ Profundus and trigeminal nerves are fused in agnathans, some teleosts and sarcopterygians, however, they are separated in cartilaginous and some ray-finned fishes.^9^^,^^10^ These differences could be related to the living environments of vertebrates especially fishes.

Overall 22 cranial nerves are distinguished in most fishes.^7^^,^^9^^,^^10^ Some of the nerves are sensory, others are motor or mixed nerves. The four nerves of Te, I, II and Ep nerves are sensory and located in the forebrain.^7^

The III and IV nerves are located in the midbrain and are motor nerves. The remaining sensory, motor and mixed nerves are correlated with the hindbrain, including VII, VIII, IX and X nerves.^7^ Researches of the developmental of brain and nerves provide key informations on the relations of the difference structures.^10^

A group of neuron cells form bipolar sensory neurons during developing neural tube, and are located in neural crest. These neurons also form the sensory ganglion of Te nerve, I, Pro, V, VII, VIII, IX, X and lateral line nerves.^11^

The Te nerve was first described in cartilaginous fishes in 1905.^5^ This nerve is a bipolar free nerve end and located in the nasal epithelium and olfactury bulb diffusely. Its modality is not detected as yet. The Te nerve derives from the olfactory placode and projects to the areas in ventral forebrain and only in the ray-finned fishes projects to the retinal region.^10^

Many of its fibers, cell bodies contain a hormone, named luteinizing-hormone releasing hormone (LHRH) or gonadotropin-releasing hormone (GnRH), that controls the production of the sex hormones and affect reproductive behaviors.^11^

The olfactory nerve (I) is created from the efferent fibers of the olfactory bipolar cells. This nerve is observed in this fishes with the sessile olfactory bulb and its function is received by smell senses.^11^ Nerve I derives from the olfactory placode and translates olfactory information to the olfactory bulb. This nerve also has an important role in detection of chemical factors.^12^^, ^^13^

The optic nerve (II) arises from retinal neurons and is a sensory nerve. This nerve in the pelagic fishes is large and thick. The retinal bipolar neuron cells derive from neural tube rather than the neural crest and placode.^14^ Nerve II courses caudally and medially from the surface of orbit in all fishes. Optic nerves cross together in the optic chiasm and in this area the axons are decussated to the adverse side.^14^

Epiphysial nerve (Ep), a sensory nerve is a second vision nerve in diencephalon jawed fishes. Ep nerve originates from epiphysial nerve cell bodies which obtain visual informations from photoreceptor cells in the pineal gland. This nerve regulates the dark-light circadian cycle,^15^ which were not distinguished up to 54-days in *H. huso* in this study.

Oculomotor nerve (III) originates the midbrain in nearly the ventral surface of midline and is motor for extraocular muscles and parasympathetic for intraocular muscles.^16^ The nerve III only contains efferent fibers. ^17^ The oculomotor complex is usually compared to some relation nuclei as oculomotor nucleus.^17^ In the fishes similar to stargazer Astroscopu, in part of the extraoular muscles (the roof of skull muscle) it has an important structure such as electrical organ, thus the oculomotor nucleus contains oculomotor and electromotor parts.^18^ The oculomotor nerve fibers end in the extraocular muscles for regulation of eye movements. The electromotor nerve fibers end in the electrical organ and control related structures to this organs. This process is observed in adult sturgeons.^18^ The intraocular muscles of function control the eye structures and innervate parasympathetic nerve fibers and rotates eye to the upward and outward.^19^According to the previous reports the optic and oculomotor nerves were not observed in the sea lamprey up this age. They appear later in sea lamprey than in other vertebrates. The late development of the eye-related nerves in lamprey appears to be related to the late development of the eye.^19^

Trochlear nerve (IV) originates from the dorsal surface of midbrain, however in the initial stages of growth have ventro-rostral position. This nerve innervate superior oblique muscles of the eye on the opposite side,^20^ and is detected on 6-day-old *H. huso* larvae. Nerve IV innervates the contralateral superior oblique muscle in fishes. This nerve also can rotate eye to the downward and outward.^21^

The Pro nerve was a very small somatosensory component for upper face region. It exited from rostral part of the hindbrain and was located toward snout and is detected on 54-day-old fish. This nerve may correspond to deep ophthalmic part of the trigeminal nerve in the most vertebrates.^22^ Profundus and trigeminal ganglion separate in cartilaginous and some ray-finned fishes, however, in agnathans, some teleosts and sarcopterygian radiations are fused.^23^ In Crossopterygii, Pro innervates the skin of snout and rostral part of tubular organs mucosal walls in which ateral line receptors are located.^23^

Trigeminal nerve (V) appears in 6-day-old fish and course bilaterally from caudo-ventral hindbrain and is located rostrally. It contains somatosensory component for face region and motor components for visceral arch muscles.^4^ Somatosensory components contains afferent and efferent nerve fibers in fishes. The afferent fibers are sensitive to the magnetic pulses specially in some teleost.^24^ Trigeminal afferent fibers terminate to some sensory nuclei in the rostro-caudal part of the brain.^25^ Efferent fibers innervate the respiratory system and support breathing action.^26^ The motor component innervates the visceral arch and muscles of mandible in teleost fishes.^4^ In lampreys, it innervates the mandibular muscles for feeding in the sucking mouth and rasping organ.^5^ In fighting fish as Betta splendens, the trigeminal nerve innervates the dilator operculi muscle used for aggressive opercular demonstration behavior.^27^ Trigeminal nerve has two branches in lampreys and sturgeon, however, it has a single origin in sharks and teleosts.^27^

The LLn contains ALLn and PLLn. The LLn is observed bilaterally in rostro-dorsal, rostro-ventral and inner otic area on 6-day-old fish. The PLLn is located in the dorso-caudal part of vagal lobe and detected on 54-day-old fish. This nerve includes about six nerves in fishes: Antero-dorsal, antero-ventral, otic, middle, supratemporal and posterior.^27^ Lateral line nerves originate from the hindbrain and in this area form the lobes in some teleosts.^5^ These nerves are mechano-sensory system of all fishes and are used in the recognition of water movements and also innervate the electro-sensory receptors.^28^

Facial nerve (VII) is located in rostro-ventral part of the medulla oblongata and observed in 54-day-old fish. Its function is gustatory and mixed. Motor component innervates visceral arch muscles in dorsal part and sensory component innervates the anterior oral cavity, lips and body surface and is responsible for gustatory sense in ventro-lateral part.^29^

Octaval nerve (VIII) is formed the dorso-caudal part of the facial-octaval ganglion complex and originats from hindbrain on 54-day-old fish. This nerve is mixed and is classified as specially somatic afferent which project to the octaval nuclei in hindbrain.^6^ It innervates the inner ear structures which contains semicircular chanels that recognize angular velocity, the utriculus that distinguishes displacement caused by gravity and sacculus and lagena that recognize displacement caused by sound waves.^30^

Vagus nerve (X) is a complex nerve with some branches and situated in caudal part of hindbrain and observed on 6-day-old fish. Vagus nerve is mixed and contains gustatory (sensory) and motor components. Gustatory nerve fibers innervate the branchial arch region, specially epibranchial organ in osteoglossid fish,^31^ also support tactile and proprioceptive innervation to the similar respective pharyngeal areas.^32^ The motor component nerve is located in dorsal area and innervate visceral arch muscles and appear from the lateral part of hindbrain. It is related to the visceral cavities as heart, gastrointestinal tract, swim bladder and pneumatic duct.^33^ In cyprinidae vagal motor nerve innervate the muscles of palatal structure, muscular structure in the roof of mouth, and gill rakers.^34^ The cranial nerves in vertebrates are responsible for different behaviors, including olfaction, vision, taste, balance and eye movements.^34^ The vagus nerve has two peripheral branches in larval lampreys.^34^

With respect to above studies of the embryological development of the nerves, it seems that in *H. huso* larvae, the activities are mostly depended on the nerves development that provide key information on the interrelationship of the different structures of the body as ear, lateral line organ on *H. huso* larvae. We suggest to study cranial nerves development on the other species and compare the point in the time of growth of the nerves.

## References

[1] Kimley AP, Beavers SC, Curtis TH (2002). Movements and swimming behavior of three species of sharks in La Jolla Canyon, California. Environ Biol Fish.

[2] Bemis WE, Findeis EK, Grande L (1997). An overview of Acipenseriformes. Environ Biol Fish.

[3] Northcutt RG (1996). The agnathan ark: The origin of craniate brains. Brain Behav Evol.

[4] Song J, Boord RL (1993). Motor components of the trigeminal nerve and organization of the mandibular arch muscles in vertebrates: Phylogenetically conservative patterns and their ontogenetic basis. Acta Anatomica.

[5] Nieuwenhuys R, Nieuwenhuys R, Ten Donkelaar HJ, Nicholson C (1998). Chondrostean fishes. The central nervous system of vertebrates.

[6] Noden DM (1991). Vertebrate craniofacial development: The relation between ontogenetic process and morpho-logical outcome. Brain Behav Evol.

[7] Carter GS (1967). Structure and habit in vertebrate evolution.

[8] Birstein VJ, Doukakis P, Sorkin B (1998). Population aggregation analysis of three caviar-producing species of sturgeons and implications for the species identification of black caviar. Conserv Biol.

[9] Butler AB, Hodos W (1996). Comparative vertebrate neuro-anatomy, evolution and adaptation.

[10] Northcutt RG, Butler AB (1993). The diencephalon and optic tectum of the longnose gar, Lepisosteus osseus (L): cytoarchitectonics and distribution of acetylcho-linesterase. Brain Behav Evol.

[11] Springer AD (1983). Centerifugal innervation of goldfish retina from ganglion cells of the nervus terminalis. J Comp Neurol.

[12] Demski LS, Northcutt RG (1983). The terminal nerve: A new chemosensory system in vertebrates. Science.

[13] Nauta WJH, Feirtag M (1986). Fundamental neuroanatomy.

[14] Springer AD, Landreth GE (1977). Direct ipsilateral retinal projections in goldfish (Carassius auratus). Brain Res.

[15] Ekstrom P (1987). Photoreceptors and CSF-contacting neurons in the pineal organ of a teleost fish have direct axonal connections with the brain. J Neurosci.

[16] Szekley G, Matesz C (1993). The efferent system of cranial nerve nuclei: A comparative neuromorphological study. Adv Anat Embryol Cell Biol.

[17] Pombal MA, Rodicio MC, Anadon R (1996). Secondary vestibuo-oculomotor projections in larval sea lamprey: anterior octavolateral nucleus. J Comp Neurol.

[18] Sparks DL (2002). The brain stem control of saccadic eye movements. Nat Rev Neurosci.

[19] Metzner W (1999). Neural circuitry for communication and jamming avoidance in gymnotiform fish. J Experimental Biol.

[20] Butler AB, Ostrander GK (2000). Nervous system: Microscopic functional anatomy. Handbook of laboratory Animals: Fish.

[21] Wathey JC (1988). Identification of the teleost Edinger-Westphal nucleus by retrograde horseradish peroxidase labelling by electrophysiological criteria. J Comp Physiol.

[22] Northcutt RG, Hodgson ES, Mathewson RF (1978). Brain organization in the cartilaginous fishes. Sensory biology of sharks, skates and rays.

[23] Northcutt RG, Bemis WE (1993). Cranial nerves of the coelacanth, Latimeria chalumnae [Osteichthyes: Sarcopterygii: Actinistia], and comparisons with other craniata. Brain Behav Evol.

[24] Walker MM, Diebel CE, Haugh CV (1997). Structure and function of the vertebrate magnetic sense. Nature.

[25] Puzdrowski RL (1988). Afferent projections of the trigeminal nerve in goldfish. J Morphol.

[26] Gorlick DL (1989). Motor innervation of respiratory muscles and an opercular display muscle in Siamese fighting fish Betta splendens. J Comp Neurol.

[27] Springer AD (1983). Centerifugal innervation of goldfish retina from ganglion cells of the nervus terminalis. J Comp Neurol.

[28] Zakon HH, Atema J, Fay RR, Popper AN (1988). Adaptations for passive electroreception. Sensory Biology of Aquatic Animals.

[29] Finger TE (1993). What's so special about special visceral?. Acta Anat.

[30] Popper AN, Fay RR (1993). Sound detection and processing by fish: Critical review and major research questions. Brain Behav Evol.

[31] Braford MR (1986). A spiral center for taste in the brain of the teleost fish, Heterotis niloticus. Science.

[32] Mauri T, Caprio J (1982). Topographical organization of taste and tactile neurons in the facial lobe. Brain Res.

[33] Hornby PJ, Demski LS (1988). Functional-anatomical studies of neural control of heart rate in Goldfish. Brain Behav Evol.

[34] Morita Y, Finger TE (1987). Topographic representation of the sensory and motor roots of the vagus nerve in the medulla of goldfish. J Comp Neurol.

